# Danger of Repressing Skin Diseases

**Published:** 1848-10

**Authors:** 


*Danger of Repressing Skin Diseases.*—Several instances have occurred in the practice of M. Devergie, illustrative of the danger which may arise from the repression of chronic cutaneous affections. The reporter of the cases sums up as follows: 1. The functional disturbance of the internal organs occurs simultaneously with the subsidence of the skin disease. 2. The severity of the symptoms is proportionate to the extent and severity of the skin disease. 3. The symptoms cease on the return of the cutaneous irritation. 4. Death may occur more rapidly than from similar internal disease produced by other causes. 5. If the internal disease be treated antiphlogistically, death is precipitated, and a fatal result always ensues if the eruption cannot be restored, or an artificial one excited.—*Lon. Med. Gaz., from Gaz. des Hopitaux.*

